# Thermosensitive In Situ Ophthalmic Gel for Effective Local Delivery and Antifungal Activity of Ketoconazole Nanoparticles

**DOI:** 10.3390/gels11010013

**Published:** 2024-12-27

**Authors:** Chutima Chaiwut, Sarin Tadtong, Puriputt Akachaipaibul, Jutamas Jiaranaikulwanitch, Sudarshan Singh, Siriporn Okonogi, Dwi Marlina Syukri, Chuda Chittasupho

**Affiliations:** 1 Master’s Degree Program in Pharmaceutical Sciences, Faculty of Pharmacy, Chiang Mai University, Chiang Mai 50200, Thailand; chutima.c@cmu.ac.th; 2 Faculty of Pharmacy, Srinakharinwirot University, Nakhonnayok 26120, Thailand; sarin@g.swu.ac.th (S.T.); puriputt.akachaipaibul@g.swu.ac.th (P.A.); 3 Department of Pharmaceutical Sciences, Faculty of Pharmacy, Chiang Mai University, Chiang Mai 50200, Thailand; jutamas.jia@cmu.ac.th (J.J.); sudarshansingh83@hotmail.com (S.S.); 4 Office of Research Administration, Chiang Mai University, Chiang Mai 50200, Thailand; 5 Center of Excellence in Pharmaceutical Nanotechnology, Faculty of Pharmacy, Chiang Mai University, Chiang Mai 50200, Thailand; okng2000@gmail.com; 6 Department of Microbiology, Faculty of Medicine, Malahayati University, Lampung 35153, Indonesia; dmarlinas79@gmail.com

**Keywords:** ophthalmic preparation, in situ gel, antifungal drugs, extemporaneous preparation

## Abstract

Fungal keratitis is a severe ocular infection caused by pathogenic fungi, leading to potential vision loss if untreated. Current antifungal treatments face limitations such as low solubility, poor corneal penetration, and limited therapeutic options. This study aimed to develop a thermosensitive in situ gel incorporating ketoconazole nanoparticles (NPs) to enhance drug solubility, stability, and antifungal activity. Ketoconazole NPs were prepared using the solvent displacement method, achieving a particle size of 198.25 ± 27.51 nm, encapsulation efficiency of 94.08 ± 0.51%, polydispersity index of 0.42 ± 0.08, and a positive zeta potential value of +10.08 ± 0.19 mV. The NPs exhibited sustained zero-order release kinetics. The optimized NPs were incorporated into a poloxamer-based in situ gel, demonstrating a gelation temperature of 34.67 ± 0.58 °C and the shortest gelation time. The formulation provided a 5-fold increase in solubility and a 10-fold improvement in drug release compared to pure ketoconazole. Stability studies confirmed the gel retained its physicochemical and rheological properties for three months under various storage conditions. The in situ gel showed sustained release, effective antifungal activity against *Malassezia furfur*, and good tolerability, suggesting it as a promising alternative for treating fungal keratitis with improved bioavailability and patient compliance.

## 1. Introduction

Fungal keratitis is a severe ocular infection that can lead to vision loss or blindness, commonly caused by filamentous fungi, including *Malassezia furfur*, *Aspergillus* spp., and *Fusarium* spp., and non-filamentous fungi such as *Candida* spp. [1]. Generally, commercial antifungal eye drops, particularly those in the azole group, are often unavailable in hospitals, prompting requests for hospital pharmacists to compound these medications. However, extemporaneous preparation of eye drops carries risks, including formulation failure, as most hospital-made preparations in Thailand lack validation and supporting data on activity and stability.

Pharmacists are often tasked with preparing extemporaneous products for off-label use in hospitals when suitable commercial products are unavailable or in short supply. These preparations may be administered topically, orally, or via injection in forms such as solutions, suspensions, ointments, or injections. However, extemporaneous compounding carries significant risks, including formulation failure, where many hospital-made preparations lack pharmacological activity and stability data [2]. Formulation failures can result in incorrect dosing, toxicity, or therapeutic failure, often due to physical and chemical incompatibilities or degradation of drugs and excipients.

While natamycin, a polyene antifungal, is the only FDA-approved drug for ocular fungal keratitis, it is limited to treating superficial infections, has poor corneal penetration, and is less effective against *Candida* species and deep fungal corneal infections [3] Consequently, other antifungals, including amphotericin B, ketoconazole, miconazole nitrate, fluconazole, and echinocandins, are frequently used due to their broad-spectrum activity and lower resistance rates. Effective treatment of ocular fungal keratitis depends on the drug’s antifungal spectrum, contact time with the infected tissue, and concentration at the target site [4]. The physicochemical and pharmacokinetic properties of antifungal drugs play a crucial role in this regard.

Several strategies have been used for solubility and bioavailability enhancement. However, each method has various limitations based on the approach used. Crystal engineering faces challenges with drug and polymer miscibility, excipient compatibility, and physical instability during storage. Chemical modification methods, such as pro-drug formation, are hindered by complex screening processes, potential chemical instability, and disruption of crystallinity. Particle size reduction can lead to physicochemical stability issues and requires careful handling due to bulkiness. Amorphization offers improved solubility but is prone to recrystallization and requires specific drug–polymer miscibility. Solvent composition strategies, such as pH adjustment and co-solvents, may destabilize drug formulations over time or cause precipitation [5]. These limitations highlight the need for a tailored solution depending on the specific drug and formulation requirements.

Ketoconazole is an imidazole antifungal agent that also exhibits some anti-bacterial activity. Ketoconazole possesses a five-membered ring structure with two nitrogen atoms. The mechanism of its antifungal activity is the inhibition of ergosterol synthesis through the blockade of 14-alpha-demethylase [6]. The effectiveness of ketoconazole for treating fungal eye infections was reported by Maĭchuk et al. Ketoconazole was used to treat 29 patients with mycotic canaliculitis, conjunctivitis, keratomycosis, and endophthalmitis. Patients took a daily 200 mg tablet for 2–3 weeks, and in severe cases of keratomycosis, an additional ketoconazole emulsion was applied as eye drops. Nineteen patients were cured, five showed improvement, and five had no response [7]. No side effects were reported during the study. Ahmed et al. developed an in situ gel formulation that was non-irritating to the cornea and allowed the vesicles to penetrate deeper into the eye without toxicity, making it a promising treatment for deep fungal eye infections [8]. The study by Zhang et al. shows that a ketoconazole–cyclodextrin complex significantly increased ketoconazole concentrations in the aqueous humor compared to ketoconazole suspension and enhanced ocular bioavailability [9]. Tavakoli et al. and Chaudhari et al. revealed no irritation or vessel injury on a hen’s egg chorioallantoic membrane surface. Additionally, the modified Draize test showed that the formulation was well tolerated in rabbit eyes over 24 h [10,11].

Lipid nanoparticles (NPs) have garnered significant attention for their ability to effectively load and release drugs, particularly those from the Biopharmaceutical Classification System (BCS) classes II (low solubility and high permeability) and IV (low solubility and low permeability). Lipid NPs are especially beneficial for poorly water-soluble drugs due to lipid properties such as high solubilizing capacity, flexibility, and biodegradability, which enhance the drugs’ bioavailability [12]. Additionally, lipids as carriers can improve drug delivery, both orally by enhancing gastrointestinal solubilization and absorption and through topical application [13].

In situ gel formulations offer several advantages for ocular drug delivery, including prolonged residence time, sustained drug release, and improved patient compliance due to reduced dosing frequency [14]. The gelation temperature is a critical parameter, as it determines the temperature at which the liquid formulation transitions into a gel upon contact with the eye. An ideal gelation temperature for ophthalmic applications is close to physiological temperature (32–35 °C) to ensure gel formation occurs upon instillation without premature gelling at room temperature [15].

Numerous studies have explored the integration of nanoparticle-based drug delivery systems with in situ gels to enhance therapeutic efficacy, particularly in ophthalmic applications. Sharma et al. incorporated brimonidine tartrate nanoparticles into thermosensitive in situ gels, significantly improving drug residence time and reducing intraocular pressure in glaucoma models [16]. Rawat et al. optimized dual-sensitive gels containing nebivolol polymeric nanoparticles, leveraging thermo- and ion sensitivity to achieve prolonged pharmacokinetics and pharmacodynamics [17]. Abbas et al. applied thermo-responsive in situ gels loaded with nanoparticles to treat bacterial keratitis, demonstrating sustained antibiotic release and improved therapeutic outcomes [18]. Wadetwar et al. utilized pH-sensitive in situ gels containing solid lipid nanoparticles of bimatoprost, achieving prolonged drug retention and reduced side effects in glaucoma management [19]. These studies underscore the critical role of polymers like poloxamer and carbopol, valued for their thermosensitive, mucoadhesive, and biocompatible properties, in advancing the effectiveness of ocular drug delivery systems.

This study aimed to develop an ophthalmic in situ gel containing ketoconazole NPs to enhance the drug’s physicochemical properties and antifungal activity. The goal was to create an extemporaneous antifungal in situ gel with proven characteristics and stability as an alternative treatment when commercial products are unavailable. The hypothesis was that nanotechnology, combined with solubilizing techniques, can be effectively applied by hospital pharmacists to prepare this ophthalmic in situ gel.

## 2. Results and Discussion

### 2.1. Particle Size, Polydispersity Index, and Zeta Potential of Ketoconazole-Loaded and Blank NPs

The particle size, PDI, and zeta potential of ketoconazole-loaded NPs coated with 0.1% poloxamer 407 were determined using dynamic light scattering. Following drug encapsulation, the average size of ketoconazole-loaded NPs was 198.25 ± 27.51 nm, while the blank NPs measured 171.90 ± 0.80 nm. The increase in particle size upon ketoconazole encapsulation suggests that drug loading contributed to the overall volume of the NPs, which might be due to ketoconazole either occupying the NP interior or associating with the nanoparticle surface. The polydispersity index of ketoconazole-loaded NPs was found to be 0.42 ± 0.08, indicating moderate size distribution heterogeneity, whereas the blank NPs exhibited a lower PDI of 0.229 ± 0.019. The higher PDI in ketoconazole-loaded NPs may suggest increased size variability, which could affect colloidal stability. In terms of zeta potential, ketoconazole-loaded NPs displayed a positive value of +10.08 ± 0.19 mV, whereas blank NPs showed a higher negative zeta potential of −26.3 ± 1.9 mV. The shift to a positive charge in ketoconazole-loaded NPs resulted from ketoconazole’s interaction with the NP surface, as the drug contains imidazole and piperazine rings with pKa values of 6.4 and 2.3, respectively, rendering it positively charged under physiological conditions [20]. These positive charges may promote adsorption onto the NP surface, altering the surface charge. Positive zeta potential is favorable for cellular uptake, especially with negatively charged cell membranes, and it may reduce aggregation, contributing to greater stability in biological environments [21].

The solvent displacement method is well suited for industrial-scale production of nanoparticles because this method does not require complex instrumentation, making it cost-effective and adaptable for large-scale manufacturing. By utilizing continuous-flow systems and inline mixing equipment, the method can be easily scaled up while maintaining consistency in particle size and encapsulation efficiency [22]. In addition, this method is cost-efficient due to its reliance on simple equipment and readily available solvents and excipients, such as ethanol and phosphatidylcholine. The incorporation of poloxamer 407 and other polymers, which are commercially available and affordable, further supports economic scalability. Additionally, the in situ gel’s prolonged drug release reduces the need for frequent applications, potentially lowering overall treatment costs by minimizing wastage and improving therapeutic outcomes.

### 2.2. Encapsulation Efficiency of Ketoconazole in NPs

The encapsulation efficiency of ketoconazole in the NPs was 94.08 ± 0.51%, indicating that the formulation process effectively incorporated the drug into the nanoparticle matrix. High encapsulation efficiency is critical for sustained drug release and enhances the therapeutic potential by ensuring adequate drug loading within the carrier system.

### 2.3. Physical Stability of Ketoconazole NPs

**Figure 1:**
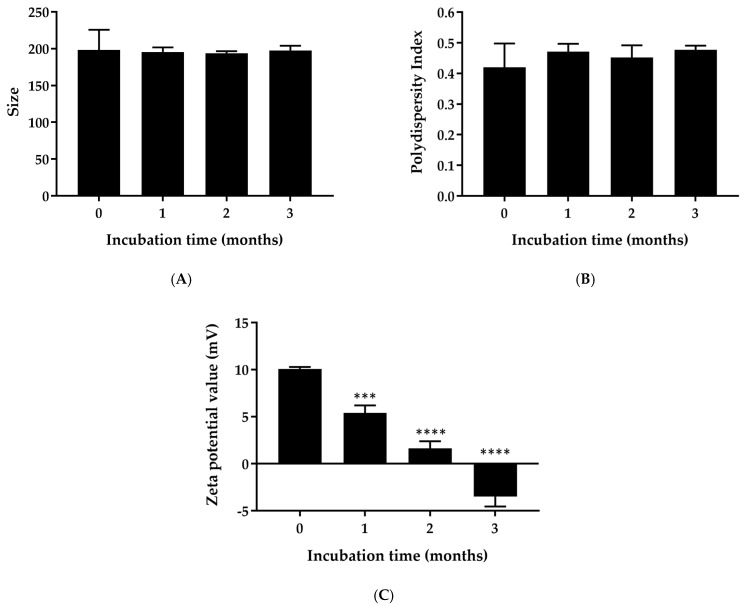
Physical stability of ketoconazole-loaded NPs (NPs) over a 3-month storage period at 30 °C. (**A**) Particle size; (**B**) PDI; (**C**) zeta potential (*** indicates *p* < 0.001 and **** indicates *p* < 0.0001).

To evaluate the physical stability of ketoconazole NPs, particle size, polydispersity index, and zeta potential were measured at 0, 1, 2, and 3 months post preparation, with NPs stored at 30 °C. The results demonstrated that ketoconazole NPs remained physically stable under these conditions, with an average particle size consistently below 200 nm and no signs of aggregation observed throughout the study (Figure 1A).

After 3 months, the PDI was recorded at 0.477 ± 0.014, within the acceptable range for particle size distribution (Figure 1B), indicating a stable particle size and distribution. This stability was due to the poloxamer coating on the NP surface, which minimizes aggregation through both electrostatic repulsion and steric stabilization, contributing to long-term stability in aqueous suspension.

A shift in zeta potential was observed over the 3-month storage period, moving to a more negative value by the end of the study (Figure 1C). This shift may suggest a gradual release of ketoconazole from the NP surface, which could alter the surface charge. Despite this change, the zeta potential remained at approximately −3.5 mV. Although a low zeta potential might typically indicate a risk for aggregation due to weaker repulsive forces, the particles remained stable in terms of size and PDI. This stability might be attributable to the dual action of the poloxamer coating, which not only provides steric hindrance but also enhances electrostatic stabilization, even with a mildly negative surface charge [23]. These findings suggest that poloxamer-coated ketoconazole NPs can maintain physical stability for at least 3 months at room-temperature storage conditions.

### 2.4. Encapsulation Efficiency of Ketoconazole NPs

The encapsulation efficiency of the prepared ketoconazole NPs was assessed using UV–vis spectrophotometry, as described in the Section 4.2. The optimized formulation exhibited a high encapsulation efficiency of 94.08 ± 0.51%, indicating successful and efficient encapsulation of ketoconazole within the nanoparticle matrix. This high encapsulation efficiency reflects the effectiveness of the formulation process in incorporating the drug, which is critical for achieving sustained release and enhanced therapeutic potential of the NPs. The encapsulation of ketoconazole was based on phosphatidylcholine. Phosphatidylcholine has a strong safety profile when used in subcutaneous injections [24]. Systemic adverse effects, such as diarrhea, nausea, or dizziness, are rare and usually mild. Long-term toxicity studies revealed no significant acute or chronic toxicity, mutagenicity, or teratogenicity. It has also been safely used in high doses for other medical applications, such as liver treatment and neonatal respiratory distress syndrome.

### 2.5. In Vitro Release of Ketoconazole from NPs

**Figure 2:**
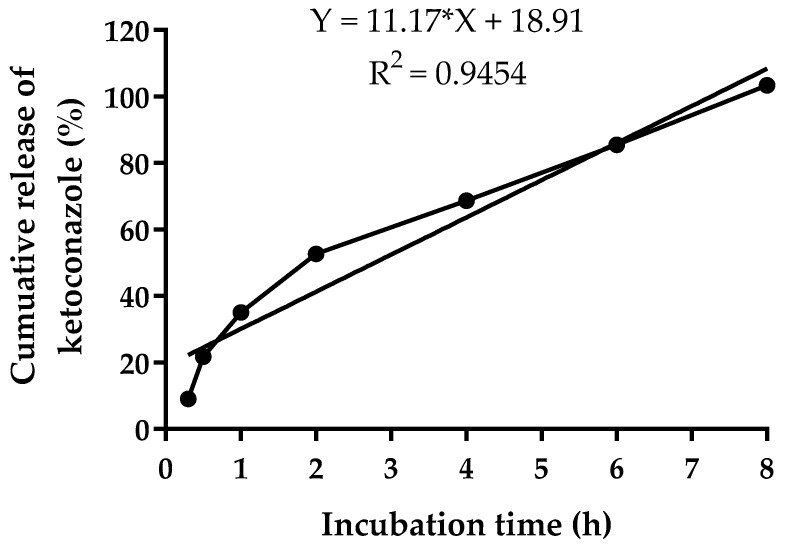
Cumulative release profile of ketoconazole from NPs over an 8 h incubation period in phosphate-buffered saline (pH 7.4) at 33 ± 0.5 °C.

The release profile of ketoconazole from NPs under sink conditions is presented in Figure 2. Approximately 35.08 ± 1.87% of ketoconazole was released within the first hour. This rapid release suggests the diffusion of ketoconazole molecules adsorbed on the NP surface into the surrounding hydrophilic shell, which facilitates their quick dispersion into the release medium. The amount of ketoconazole released was up to 103.4 ± 1.8% over 8 days at pH 7.4.

The release kinetics of ketoconazole from the NPs aligned with a zero-order kinetic model (R^2^ = 0.9454), indicating a consistent release rate independent of drug concentration over time. The initial release may reflect the desorption of surface-bound ketoconazole, while the controlled-release phase indicates effective entrapment of the drug within the nanoparticle core. This release profile is desirable for maintaining steady drug concentrations in the eyes, reducing dosing frequency, and improving therapeutic outcomes.

### 2.6. Kinetic Degradation of Ketoconazole in NPs

**Figure 3:**
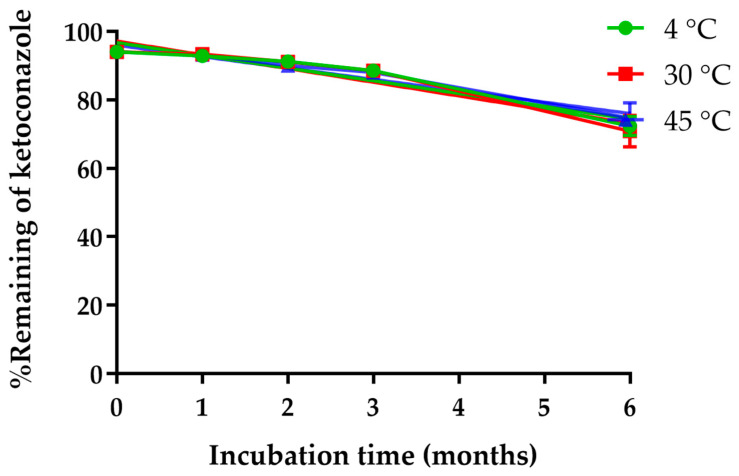
Chemical stability of ketoconazole-loaded NPs over a 3-month storage period at three different temperatures (4 °C, 30 °C, and 45 °C).

The stability of ketoconazole encapsulated in the NPs, prepared via a solvent displacement method, was evaluated over a 3-month period at storage temperatures of 4 °C, 30 °C, and 45 °C. As shown in Figure 3, the percentage of ketoconazole remaining within the NPs decreased gradually over time at all temperatures, with final values after 3 months of 88.54 ± 1.57% at 4 °C, 88.02 ± 0.36% at 30 °C, and 88.52 ± 1.08% at 45 °C. These results indicate a steady and nominal degradation of ketoconazole within the phospholipid matrix over the time tested, regardless of storage temperature. The degradation of ketoconazole in the NPs followed a zero-order kinetic model, as evidenced by the linear relationship between ketoconazole concentration and time, with high correlation coefficients (R^2^ = 0.9666, 0.9487, and 0.9626 for 4 °C, 30 °C, and 45 °C, respectively). This zero-order kinetics suggests that ketoconazole degradation proceeded at a constant rate independent of its concentration, where degradation may be influenced by the lipid structure and encapsulation method rather than temperature. The zero-order release kinetics demonstrated by the ketoconazole-loaded nanoparticles incorporated into the in situ gel ensure a consistent release rate, independent of drug concentration. This steady release reduces the risk of drug peaks and troughs, thereby avoiding subtherapeutic levels that could lead to fungal resistance and treatment failure. In addition, sustained release minimizes the need for frequent dosing, which is particularly advantageous for improving patient compliance in chronic treatments. The rate constants calculated for each temperature were 1.83, 1.90, and 2.13% per month at 4 °C, 30 °C, and 45 °C, respectively, supporting this finding, as they remained relatively consistent across the tested storage conditions. The calculated shelf life (t_90_), or the time required for 90% of the initial ketoconazole to remain in the formulation, was approximately 5.2 months at 4 °C, 5.0 months at 30 °C, and 4.4 months at 45 °C. These similar t₉₀ values further suggest that temperature had a minimal effect on ketoconazole stability within the lipid NPs.

Ketoconazole undergoes degradation primarily under acidic, basic, and oxidative stress conditions [25]. Acidic and basic hydrolysis result in a demethylated degradation product, formed by the cleavage of the ketoconazole structure. Oxidative degradation produces an N-oxide derivative, caused by the oxidation of the nitrogen atom in the piperazine ring, introducing an additional oxygen atom [26]. The efficacy and safety of ketoconazole degradation products might be unaffected, as the pharmacophore of intact ketoconazole, including the imidazole ring essential for inhibiting fungal ergosterol biosynthesis, remains intact following hydrolysis and oxidation.

### 2.7. Antifungal Activity of Ketoconazole NPs

The antifungal activity of free ketoconazole and ketoconazole NPs was evaluated against *Malassezia furfur* at various concentrations, as summarized in Table 1. Antifungal activity was determined based on observable growth inhibition. Each concentration and treatment condition were tested in triplicate to ensure accuracy and reproducibility.

Free ketoconazole demonstrated effective antifungal activity at higher concentrations of 20 µg/mL, 10 µg/mL, and 5 µg/mL, with significant growth inhibition (“-”) observed consistently across all strains and replicates. This suggests that free ketoconazole effectively inhibits *M. furfur* at these concentrations. At a moderate concentration of 2.5 µg/mL, free ketoconazole also displayed consistent antifungal activity, with all replicates showing complete inhibition across the three experiments. At the lowest concentration tested (1.25 µg/mL), free ketoconazole showed inconsistent antifungal activity. Only one replicate in strain N3 displayed slight fungal growth (“+”), while the remaining replicates showed significant growth inhibition (“-”). These results suggest that 2.50 µg/mL was considered as a minimum effective concentration for antifungal activity with free ketoconazole.

Ketoconazole NPs exhibited significant antifungal activity (“-”) at concentrations of 20 µg/mL, 10 µg/mL, and 5 µg/mL across all experiments and replicates, similar to the results seen with free ketoconazole. This indicates that encapsulating ketoconazole within NPs does not reduce its antifungal efficacy at higher concentrations. At 1.25 µg/mL, ketoconazole NPs demonstrated minimal and inconsistent antifungal activity, with only one replicate showing slight fungal growth (“+”). This is consistent with the results for free ketoconazole, suggesting that 2.50 µg/mL is the minimum effective concentration threshold for reliable antifungal activity, whether in free or NP form.

The control groups, including 1% DMSO/medium, plain medium, and blank NPs (without ketoconazole), consistently showed normal fungal growth (“++”) across all replicates and experiments, indicating no antifungal activity. This confirms that any observed antifungal effects are attributable solely to ketoconazole or ketoconazole-loaded NPs.

Both free ketoconazole and ketoconazole-loaded NPs demonstrate effective antifungal activity against *M. furfur* at concentrations of 2.5 µg/mL and above. The consistent inhibition observed at these concentrations suggests that ketoconazole is effective at inhibiting *M. furfur* growth in both its free and nanoparticle forms. At the lowest concentration tested (2.50 µg/mL), antifungal activity was minimal and inconsistent, suggesting that this concentration may be below the therapeutic threshold for reliable inhibition of *M. furfur*.

The comparable antifungal activity of ketoconazole NPs and free ketoconazole across all tested concentrations indicates that encapsulation in NPs does not compromise the antifungal efficacy of ketoconazole. This suggests that ketoconazole-loaded NPs could serve as an effective alternative to free ketoconazole, potentially offering additional benefits such as controlled release and targeted delivery without reducing the drug’s antifungal potency. Zhu et al. demonstrated that thermo-sensitive in situ gelling formulation of ketoconazole based on poly(N-isopropyl acrylamide)/hyaluronic acid was well tolerated by rabbits, and no macroscopic signs of irritation, redness, or other toxic effects were observed [27].

### 2.8. Gelation Temperature and Gelation Time

The gelation temperature is defined as the temperature at which a formulation transitions from a liquid to a gel state. For ophthalmic applications, an ideal gelation temperature is in the range of 32–35 °C [15]. A gelation temperature below 25 °C may lead to gel formation at room temperature, which can pose challenges in manufacturing and administration due to premature gelling. Conversely, if the gelation temperature is higher than 35 °C, the formulation would remain in a liquid state at eye temperature, leading to inadequate control over drug release and reduced retention time in the eye.

The gelation temperature, gelation time, and pH values of the in situ gel loaded with ketoconazole NPs are presented in Table 2. In this study, the gelation temperature of the in situ gel loaded with ketoconazole NPs was measured and found to be 34.67 ± 0.58 °C. This temperature falls within the ideal range for ophthalmic use, suggesting that the formulation would transition to a gel state at body temperature, ensuring controlled drug release and prolonged retention in the eye. Consequently, this gelation temperature supports the suitability of the ketoconazole NP-loaded in situ gel for ophthalmic application.

### 2.9. Effect of In Situ Gel Composition on Gelation Temperature and Gelation Time

Poloxamer 407 played a pivotal role in gelation temperature control. An increase in poloxamer 407 concentration resulted in reducing gelling temperature. Formulations with 16% poloxamer (e.g., F3) exhibited the most optimal gelation temperature (34.67 °C) compared to those containing 17% and 18% poloxamer, which gelled at 31 and 30 °C, respectively. Adding HPMC into poloxamer solution at concentrations of 0.5, 1, and 2% in F4–F6 was associated with lower gelation temperatures (<28 °C) and reduced gelation times (around 53 s). This result indicates that HPMC facilitates gel formation at lower temperatures and accelerates the gelation process. This could be attributed to HPMC’s ability to form hydrogen bonds, which enhances the gel structure at lower temperatures [28]. The presence of SCMC in formulations significantly influenced gelation properties, particularly by increasing the gelation temperature. Formulations containing SCMC, such as F13–F15, displayed gelation temperatures above 40 °C, the highest in this study. Additionally, higher SCMC concentrations correlated with extended gelation times, for instance, F14 with a gelation time of 80 s. These findings suggest that SCMC delays the onset of gelation. Sodium alginate, another gelling agent, demonstrated effects similar to HPMC. Formulations with sodium alginate, such as F18, gelled at lower temperatures (<28 °C) and had relatively shorter gelation times around 61 s. This indicates that sodium alginate aids in achieving gelation at lower temperatures and within a shorter time frame.

The pH of the formulations ranged from 6.89 to 7.17, with minimal variation across different compositions. This consistency suggests that changes in the concentrations of poloxamer 407, HPMC, SCMC, and sodium alginate do not significantly alter the pH, maintaining a near-neutral environment suitable for ketoconazole stability and compatibility with biological tissues.

Poloxamer 407 has demonstrated a strong safety profile for ocular drug delivery. Multiple studies have confirmed its biocompatibility with in vitro and ex vivo tests, such as the MTT assay and corneal erosion tests, showing non-toxicity to human and animal corneal tissues [29]. In vivo evaluations, including modified Draize tests and confocal ophthalmoscopy, revealed no significant irritation or adverse effects on the corneal surface, making them well tolerated for topical ocular use [30]. However, intravitreal applications of high-concentration poloxamer formulations (≥20%) have been associated with retinal atrophy and mild cataracts, indicating the need for caution and further research in such contexts [31,32]. Overall, poloxamers are safe and effective for anterior ocular applications, with their tolerability supporting their use in sustained drug delivery systems.

The preservatives used in this study, including methylparaben and propylparaben, are commonly employed in ophthalmic formulations to ensure microbial stability. These compounds are generally recognized as safe (GRAS) by regulatory agencies when used within recommended concentrations. Methylparaben is used in ophthalmic preparations at a safe concentration of 0.015–0.05% [33]. One study found that concentrations of propylparaben in excess of 0.5% in ophthalmic preparations can irritate the eye [33]. Alternative preservatives, such as benzalkonium chloride-free options like polyquaternium-1 or oxidative preservatives (e.g., stabilized oxychloro complexes), are known to provide antimicrobial efficacy while minimizing irritation or toxicity to ocular tissues. These options are particularly beneficial for patients with sensitive eyes or those requiring long-term use of ophthalmic products.

### 2.10. Effect of Incubation Time and Temperature on Gelation Time

**Figure 4:**
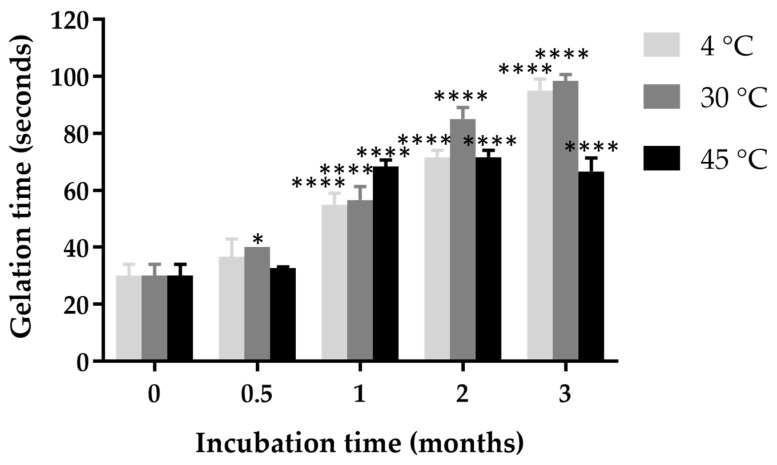
Gelation time of in situ gel loaded with ketoconazole NPs during a 3-month incubation period at different storage temperatures (4 °C, 30 °C, and 45 °C). Statistical significance compared to initial gelation time is indicated as follows: * indicates *p* < 0.05 and **** indicates *p* < 0.0001.

The gelation time of in situ gel loaded with ketoconazole NPs increased with incubation time across all temperature conditions, as shown in Figure 4. After 0.5 months of incubation, the gel stored at 4 °C exhibited a statistically significant increase in gelation time compared to those at 45 °C (*p* < 0.05). By 2 months, gelation times at 4 °C were significantly higher than those at 30 °C and 45 °C, with a marked increase (*p* < 0.001) observed for the gel stored at this lower temperature. This trend persisted and became more pronounced at 3 months, where samples incubated at 4 °C exhibited the longest gelation times, followed by those at 30 °C, while those at 45 °C had the shortest gelation times.

Poloxamer 407 is a thermo-reversible polymer, typically forming gels at higher temperatures due to micellization, where individual polymer molecules aggregate to form a gel network [34]. At 4 °C, gelation times were substantially prolonged compared to those at 30 °C and 45 °C, indicating that low temperatures may retard the structural organization or micellization of poloxamer 407, resulting in delayed gel formation. An increase in temperature facilitates the formation of micelles at lower concentrations of poloxamer 407, primarily driven by the desolvation of the polypropylene oxide core [35]. At lower temperatures, poloxamer molecules may not achieve the critical concentration and alignment required for rapid gelation, as the reduced thermal energy limits their ability to overcome activation barriers for micellization [36]. The poloxamer micelles consist of a hydrophobic inner core formed by the PPO blocks and a hydrophilic outer shell formed by the PEO units. Typically, nano-sized micelles, ranging from 10 to 200 nm, form at the critical micellization concentration and critical micellization temperature. For poloxamer aqueous solutions, the critical micelle concentration (CMC) decreases with increasing temperature and the number of PEO segments, indicating that polymers with larger hydrophobic (PPO) domains can form micelles at lower concentrations and temperatures [37]. Furthermore, this delayed gelation at low temperatures may have practical implications for the storage and stability of in situ gel loaded with ketoconazole NPs. Prolonged storage at refrigeration temperatures could potentially impact the gelation of in situ gel by taking longer times to achieve gel consistency.

Blending poloxamer 407 with HPMC, SCMC, and sodium alginate is significant because it enhances the properties of thermo-responsive gels for pharmaceutical applications. Silva et al. reported that adding HPMC or SCMC into poloxamer improves gelation temperature, rheological properties, and mechanical strength, enhancing performance in topical formulations [38]. Sodium alginate plays a significant role in polymer blends by enhancing mucoadhesive properties, lowering the sol–gel transition temperature, and improving gel retention. The addition of sodium alginate to poloxamer 407 solution promotes the dehydration of polyethylene oxide chains, facilitating gelation at lower temperatures [39].

### 2.11. Effect of Incubation Time and Temperature on Gelation Temperature

**Figure 5:**
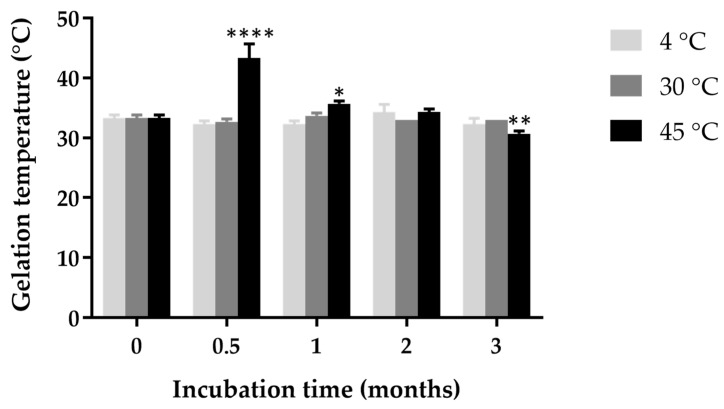
Gelation temperature of in situ gel loaded with ketoconazole NPs over a 3-month incubation period at different storage temperatures (4 °C, 30 °C, and 45 °C). Statistical significance compared to initial gelation temperature is indicated as follows: * indicates *p* < 0.05, ** indicates *p* < 0.01, and **** indicates *p* < 0.0001.

After 0.5 months of incubation, a significant increase in gelation temperature was observed in the in situ gel loaded with ketoconazole NPs stored at 45 °C compared to those stored at 4 °C and 30 °C (*p* < 0.0001) (Figure 5). This indicates that high-temperature storage may induce structural or chemical changes within the poloxamer matrix, resulting in a higher temperature requirement for gelation. The increased gelation temperature at 45 °C could be attributed to enhanced molecular interactions or partial dehydration of the poloxamer molecules, which restricts the mobility of polymer chains and hinders gel formation at lower temperatures [40]. At 1 month, a moderate increase in gelation temperature was still observed in the 45 °C samples (* *p* < 0.05). By 2 and 3 months, the gelation temperatures across all conditions stabilized, with the samples stored at 45 °C still showing a mild but statistically significant elevation in gelation temperature by the 3-month storage.

### 2.12. Effects of Incubation Time on pH

**Figure 6:**
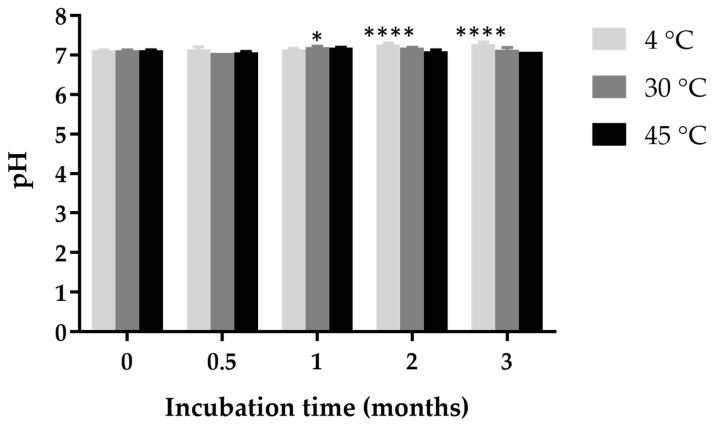
pH stability of in situ gel loaded with ketoconazole NPs over a 3-month incubation period at different storage temperatures (4 °C, 30 °C, and 45 °C). Statistical significance compared to the initial pH value is indicated as follows: * indicates *p* < 0.05, **** indicates *p* < 0.0001.

The pH values remained relatively stable across the different storage temperatures (4 °C, 30 °C, and 45 °C) and incubation times, with minimal fluctuations observed (Figure 6). While in situ gel loaded with ketoconazole NPs demonstrated good overall pH stability, a slight pH decrease at 4 °C over extended incubation periods suggests that low-temperature storage might influence pH maintenance.

### 2.13. Rheology and Viscosity of In Situ Gel Loaded with Ketoconazole NPs

**Figure 7:**
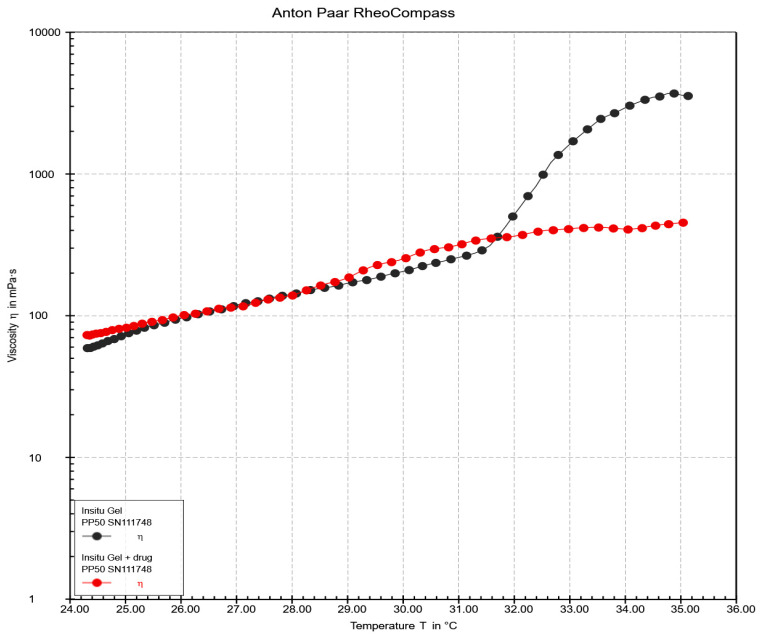
Viscosity-temperature profile of in situ gel formulations with and without ketoconazole-loaded NPs.

The viscosity profiles of the in situ gels, both with and without the drug, were evaluated as a function of temperature (Figure 7). The data demonstrate distinct trends in viscosity as temperature increases from 24 °C to 35 °C.

The in situ gel without drug incorporation showed a gradual increase in viscosity from approximately 24 °C to around 300 mPa·s at 32 °C. Beyond 32 °C, the viscosity increased sharply, reaching values above 1000 mPa·s by 35 °C. In contrast, the in situ gel loaded with ketoconazole NPs exhibited a similar gradual increase in viscosity up to 32 °C, paralleling the behavior of the drug-free gel in the lower temperature range. However, the viscosity of the in situ gel loaded with ketoconazole NPs gradually increased and did not exhibit the same sharp increase observed in the drug-free gel.

**Figure 8:**
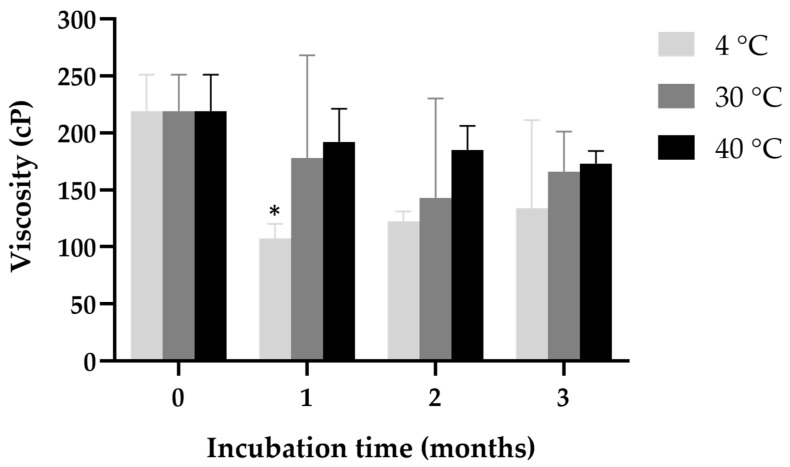
The viscosity of ketoconazole-loaded NPs stored at three different temperatures (4 °C, 30 °C, and 45 °C) over a 3-month incubation period. * indicates *p* < 0.05.

Figure 8 illustrates the viscosity of ketoconazole-loaded NPs stored at three different temperatures (4 °C, 30 °C, and 45 °C) over a 3-month incubation period. As the incubation progressed, differences in viscosity trends emerged based on storage conditions. At 4 °C, a significant reduction in viscosity occurred after 1 month, dropping to approximately 150 cP (at a shear rate of 100 s^−1^).

Conversely, at 30 °C, the viscosity showed a slight decline after 1 month but stabilized above 200 cP throughout the 3 months. This indicates that the formulation retains its rheological integrity better at this temperature, making it potentially more suitable for storage. Similarly, at 45 °C, the viscosity remained relatively stable over the 3-month period, with minor fluctuations. Despite the higher temperature, the viscosity was consistently above 200 cP, suggesting that the in situ gel loaded with ketoconazole NPs demonstrated strong thermal stability.

The thermosensitive behavior of poloxamer 407 primarily arises from its temperature-dependent hydrophobic and hydrophilic balance, which facilitates self-assembly and micelle aggregation [41]. Storage at 4 °C led to extended gelation times and decreased viscosity, which might be due to the delayed micellization and structural rearrangement of the poloxamer molecules. In contrast, storage at 30 °C and 45 °C preserved the gel’s viscosity and gelation properties, with 30 °C providing the most stable conditions. These findings highlight the importance of storage temperature in maintaining the functional properties of in situ gels.

The gel was designed with a low viscosity at room temperature, enabling it to be easily dispensed as a liquid through ophthalmic droppers. Upon contact with the ocular surface, the thermosensitive properties of the gel ensure a rapid transition to a gel state at physiological temperatures, providing prolonged retention and sustained drug release.

Although patient compliance was not directly assessed in this study, the formulation was specifically designed to enhance user convenience and compliance by reducing the frequency of application compared to conventional eye drops. The prolonged retention and reduced dosing frequency are expected to improve adherence, especially in chronic conditions of fungal keratitis.

## 3. Conclusions

This study demonstrates the successful development of ketoconazole-loaded NPs and their incorporation into an in situ gel system for ocular drug delivery. The formulation exhibits favorable physicochemical properties, high stability, controlled release, and potent antifungal activity. The gelation characteristics and rheological properties support its potential for clinical application, particularly for sustained delivery in the treatment of fungal keratitis. Further, in vivo studies are warranted to validate these findings and assess the therapeutic efficacy of the formulation under physiological conditions.

## 4. Materials and Methods

### 4.1. Materials

Ketoconazole and hydroxypropyl methylcellulose 4000 were obtained from S. Tong Chemicals Co., Ltd., Bangkok, Thailand. Phosphatidylcholine and poloxamer 407 were purchased from Chanjao Longevity Co., Ltd., Bangkok, Thailand. Sodium carboxymethyl cellulose (medium viscosity) was purchased from Sigma-Aldrich, St. Louis, MO, USA. Sodium alginate was purchased from Chemipan Corporation Co., Ltd., Bangkok, Thailand. Methylparaben and propylparaben were obtained from B.L. Hua & Co., Ltd., Bangkok, Thailand. *Malassezia furfur* ATCC14521 was purchased from the American Type Culture Collection, Manassas, VA, USA. Malt extract and peptone were obtained from Becton, Dickinson and Company, Le Pont-de-Claix, France. Desiccated ox bile was from Himedia, India. Glycerol and oleic acid were purchased from SL Quality Supply, Bangkok, Thailand. Tween 40^®^ was obtained from Merck & Co., Rahway, NJ, USA.

### 4.2. Methods

#### 4.2.1. Ketoconazole Nanoparticle Preparation and Characterization

Ketoconazole NPs were prepared using the solvent displacement method as reported [42]. Briefly, ketoconazole (100 mg) was dissolved in 2 mL of absolute ethanol, followed by the addition of phosphatidylcholine (100 mg). The resulting mixture was introduced dropwise into 10 mL of 0.1% poloxamer 407 solution at a rate of 8 mL/h. The solution was stirred for 30 min at room temperature to allow nanoparticle formation. The ketoconazole NPs were subsequently collected by centrifugation at 13,000 rpm for 10 min and washed with purified water. Blank nanoparticles were prepared using the same procedure without the addition of ketoconazole. The size, polydispersity index (PDI), and zeta potential of blank and ketoconazole-loaded NPs were determined using dynamic light scattering (Zetasizer Nano series, Malvern Instruments, Worcestershire, UK.). Multiple batches of ketoconazole nanoparticles were prepared under identical conditions. The particle size, polydispersity index (PDI), and zeta potential were evaluated for consistency.

#### 4.2.2. Determination of Encapsulation Efficiency

The encapsulation efficiency of ketoconazole NPs was determined using UV–visible spectrophotometry. After formation, 1 mL of the ketoconazole NP suspension was sampled and centrifuged at 13,000 rpm for 10 min. The resulting nanoparticle pellets were collected and dissolved in dimethyl sulfoxide (DMSO). The amount of ketoconazole was quantified by measuring absorbance at 270 nm using a SpectraMax M3 multi-mode microplate reader (Molecular Devices, San Jose, CA, USA). Encapsulation efficiency (%) was calculated using the following equation:

$$Encapsulation\;efficiency\;\left(\%\right)=\frac{Amount\;of\;ketoconazole\;in\;NPs}{Amount\;of\;ketoconazole\;at\;initial}\times 100\%$$

#### 4.2.3. In Vitro Release Study of Ketoconazole NPs

The release study of ketoconazole from NPs was conducted following a previously described protocol [43]. The release study of ketoconazole from NPs was designed to assess the drug’s release profile using a Transwell^®^ system (Corning, Glendale, AZ, USA), a commonly employed model for controlled-release studies. Ketoconazole NPs (200 µL at 10 mg/mL) were applied to a semi-permeable membrane with a 0.4 µm pore size in a Transwell^®^ insert. The receptor compartment, situated below the membrane, contained 1.0 mL of phosphate-buffered saline (PBS) at pH 7.4, preheated to 33 ± 0.5 °C to simulate physiological conditions. During the study, 100 µL samples were periodically withdrawn from the receptor compartment at intervals of 15 min, 30 min, 1 h, 2 h, 4 h, 6 h, and 8 h. Each withdrawn sample was immediately replaced with an equal volume of fresh, preheated PBS to maintain consistent sink conditions, which prevent saturation and allow continued diffusion of the drug. The amount of ketoconazole released into the receptor compartment was quantified by measuring absorbance at 270 nm using a SpectraMax M3 multi-mode microplate reader. A standard curve for ketoconazole was established to calculate drug concentrations in the receptor compartment.

$$Cumulative\;ketioconazole\;released\;\left(\%\right)=\frac{Amount\;of\;released\;ketoconazole}{Amount\;of\;total\;ketoconazole\;in\;NPs}\times 100\%$$

where the amount of released ketoconazole was determined by UV spectroscopy, and the amount of total ketoconazole in the NPs was the amount of ketoconazole encapsulated in the NPs.

#### 4.2.4. Determination of Physical Stability of Ketoconazole NPs

Ketoconazole NPs in deionized water were stored in sealed vials at 30 °C for a duration of 3 months. At predetermined time points (1, 2, and 3 months), samples were taken to assess particle stability. The particle size, PDI, and zeta potential of the samples were measured using dynamic light scattering (DLS) to evaluate any changes in physical properties over the storage period.

#### 4.2.5. Determination of Chemical Stability of Ketoconazole Entrapped in Ketoconazole NPs

Ketoconazole NPs suspended in deionized water (10 mg/mL) were stored in tightly sealed vials at 30 °C for a period of 3 months. At specified intervals (1, 2, and 3 months), samples were collected and centrifuged at 13,000 rpm for 10 min. After centrifugation, the supernatant was discarded, and ketoconazole NPs were dissolved in dimethyl sulfoxide (DMSO). The concentration of ketoconazole remaining in the NPs was determined by measuring absorbance at 270 nm using a SpectraMax M3 multi-mode microplate reader (Molecular Devices, San Jose, CA, USA). The degradation data of ketoconazole over the storage period were analyzed using various kinetic models, including zero-order, first-order, and second-order kinetics. The most suitable kinetic model for the degradation data was identified by comparing the correlation coefficient (R^2^) values for each model, allowing for the determination of the best-fit model to describe the degradation behavior of ketoconazole in the NPs under storage conditions.

#### 4.2.6. Antifungal Activity Study of Ketoconazole NPs Against *Malassezia furfur* by Broth Microdilution Assay

The assay for minimum inhibitory concentration (MIC) of ketoconazole and its NPs was performed using broth microdilution technique in a 96-well plate [44]. The assay was performed in three independent experiments (*n* = 3), and each experiment was run in triplicate. *M. furfur* was cultured in a Dixon’s medium at 28 °C for 4 days in an incubator prior to performing the assay. Then, *M. furfur* at turbidity equal to 0.5 McFarland standard solution (1 × 10^8^ CFU/mL) was made and further diluted with Dixon’s medium to obtain a 5%v/v suspension of the fungus in the medium [1]. Ketoconazole and ketoconazole NPs were diluted with 2% *v/v* DMSO in Dixon’s medium to obtain various concentrations of 2.5–40 µg/mL. Nanoparticle without ketoconazole and Dixon’s medium were used as negative control. Furthermore, 2% *v/v* DMSO in Dixon’s medium was used as solvent control. Then, 100 µL of control, solvent control, and sample were pipetted into a 96-well plate, followed by 100 µL of the fungus suspension in a medium, which gave final concentrations of tested samples that varied from 1.25 to 20 µg/mL. The plate was incubated at 28 °C for 4 days in an incubator. Then, the MIC was observed by visual detection. The lowest concentration of the tested sample that showed no fungus growth (clear solution) was the MIC.

#### 4.2.7. Preparation of In Situ Gel Loaded with Ketoconazole NPs

The in situ gel was formulated by dispersing the appropriate amounts of thermosensitive poloxamer 407, serving as the main polymer, and co-polymers including hydroxypropyl methylcellulose (HPMC), sodium carboxymethyl cellulose, and sodium alginate, into water. This dispersion was achieved using a magnetic stirrer, which was operated until the polymers completely dissolved. Subsequently, ketoconazole NPs were incorporated into the gelling solution with continuous stirring to ensure uniform distribution. A paraben concentrate (0.5 mL) was then added as a preservative to the final mixture. The compositions of the in situ gel formulations containing ketoconazole NPs are detailed in Table 3.

#### 4.2.8. Characterization of In Situ Gel Loaded with Ketoconazole NPs

##### Determination of Gelation Temperature

The gelation temperature of in situ gel loaded with ketoconazole NPs was measured by loading the gel into glass tubes and placing them in a water bath. The water bath temperature was initially set to 28 °C and increased incrementally by 1 °C. Temperature measurements were taken using a thermometer. Gelation was assessed by tilting the tube at a 90° angle. The sol-to-gel transition temperature was recorded as the lowest temperature at which the gel remained immobile upon tilting.

##### Determination of Gelation Time

The gelation time of the in situ gel loaded with ketoconazole NPs was evaluated by filling 3 mL of gel into a tube and immersing it in a water bath set at 33 ± 0.5 °C, which corresponds to the gelation temperature. The gel was monitored by inverting the tube horizontally every minute. The time at which the gel became immobile was recorded as the gelation time.

##### pH Measurement

The pH of in situ gel loaded with ketoconazole NPs was measured in triplicate at room temperature using a pH meter (Thermo Fisher Scientific, Waltham, MA, USA).

##### Rheological Measurement of In Situ Gel Containing Ketoconazole NPs

Measurement of the freshly prepared in situ gel loaded with ketoconazole NPs rheology was performed using a rheometer (Anton Paar MCR 102e Rheometer, Graz, Austria) equipped with a plate and plate geometry. The shear rate was constant at 100 s^−1^, varying temperatures from 25 to 35 °C with a heating rate of 1 °C/min. The rheogram was generated using Rheocompass software version 1.33, equipped with the instrument. The rheology and viscosity of in situ gels loaded with ketoconazole NPs after storage at 4 °C, 30 °C, and 45 °C for 1, 2, and 3 months were investigated using a Thermo Scientific HAAK E RheoStress 1 rheometer (Waltham, MA, USA), equipped with a plate and plate geometry (1.0 mm gap, 60 mm diameter). The rheological behavior and viscosity profiles of the samples were presented at a shear rate ranging from 0.1 to 200 s^−1^.

#### 4.2.9. Physical Stability Study of In Situ Gel Loaded with Ketoconazole NPs

The gelation temperature, gelation time, pH, and viscosity of freshly prepared in situ gels loaded with ketoconazole NPs were determined using the methods described above. Additionally, the physical stability of these parameters was assessed after storing the gels at 30 °C for 1, 2, and 3 months. This analysis allowed for the evaluation of the stability and consistency of the gelation properties over time and under different storage conditions.

#### 4.2.10. Statistical Analysis

Data were analyzed using one-way analysis of variance (ANOVA), followed by the Newman–Keuls method as a post hoc test to assess the significance of differences between groups (GraphPad Prism 7.02, La Jolla, CA, USA). Statistical significance was defined as * *p* < 0.05. Results are presented as mean ± standard deviation (SD) from three independent experiments (n = 3).

## Figures and Tables

**Table 1:** Antifungal activity of free ketoconazole and ketoconazole-loaded NPs (NPs) against *Malassezia furfur.*

Ketoconazole	N1			N2			N3		
	rep 1	rep 2	rep 3	rep 1	rep 2	rep 3	rep 1	rep 2	rep 3
20 µg/mL	(-)	(-)	(-)	(-)	(-)	(-)	(-)	(-)	(-)
10 µg/mL	(-)	(-)	(-)	(-)	(-)	(-)	(-)	(-)	(-)
5 µg/mL	(-)	(-)	(-)	(-)	(-)	(-)	(-)	(-)	(-)
2.5 µg/mL	(-)	(-)	(-)	(-)	(-)	(-)	(-)	(-)	(-)
1.25 µg/mL	(-)	(-)	(-)	(-)	(-)	(-)	(-)	(-)	(+)
1% DMSO/medium	(++)	(++)	(++)	(++)	(++)	(++)	(++)	(++)	(++)
Control (medium)	(++)	(++)	(++)	(++)	(++)	(++)	(++)	(++)	(++)
**Ketoconazole NPs**	**N1**			**N2**			**N3**		
	**rep 1**	**rep 2**	**rep 3**	**rep 1**	**rep 2**	**rep 3**	**rep 1**	**rep 2**	**rep 3**
20 µg/mL	(-)	(-)	(-)	(-)	(-)	(-)	(-)	(-)	(-)
10 µg/mL	(-)	(-)	(-)	(-)	(-)	(-)	(-)	(-)	(-)
5 µg/mL	(-)	(-)	(-)	(-)	(-)	(-)	(-)	(-)	(-)
2.5 µg/mL	(-)	(-)	(-)	(-)	(-)	(-)	(-)	(-)	(-)
1.25 µg/mL	(-)	(-)	(-)	(-)	(+)	(-)	(-)	(-)	(-)
1% DMSO/medium	(++)	(++)	(++)	(++)	(++)	(++)	(++)	(++)	(++)
Control (medium)	(++)	(++)	(++)	(++)	(++)	(++)	(++)	(++)	(++)
Nanoparticle	(++)	(++)	(++)	(++)	(++)	(++)	(++)	(++)	(++)

Remarks: “-” indicates complete inhibition of fungal growth, “+” indicates slight fungal growth presence, and “++” indicates normal fungal growth presence.

**Table 2:** The gelation temperature, gelation time, and pH values of the in situ gel loaded with ketoconazole NPs.

Formulation Code	Gelation Temperature (°C)	Gelation Time (s)	pH
F1	30.00 ± 0.00	63.33 ± 5.77	7.09 ± 0.01
F2	31.00 ± 0.00	70.00 ± 5.00	7.09 ± 0.01
F3	34.67 ± 0.58	63.33 ± 2.89	7.13 ± 0.03
F4	<28.00	73.33 ± 2.89	7.17 ± 0.01
F5	<28.00	51.67 ± 5.77	7.11 ± 0.01
F6	<28.00	53.33 ± 2.89	7.16 ± 0.02
F7	30.00 ± 0.00	75.00 ± 0.00	7.07 ± 0.01
F8	31.00 ± 0.00	73.33 ± 2.89	7.04 ± 0.00
F9	30.00 ± 0.00	61.67 ± 5.77	7.09 ± 0.01
F10	30.00 ± 0.00	60.00 ± 8.66	7.05 ± 0.01
F11	30.00 ± 0.00	56.67 ± 7.64	7.07 ± 0.01
F12	30.00 ± 0.00	55.00 ± 5.00	7.05 ± 0.01
F13	>40.00	76.67 ± 2.89	6.99 ± 0.01
F14	>40.00	80.00 ± 5.00	7.04 ± 0.01
F15	>40.00	78.33 ± 5.77	6.95 ± 0.00
F16	30.00 ± 0.00	60.00 ± 5.00	7.06 ± 0.01
F17	29.00 ± 0.00	63.33 ± 2.89	6.98 ± 0.01
F18	<28.00	61.67 ± 2.89	6.89 ± 0.01
F19	>40.00	86.67 ± 2.89	6.96 ± 0.01
F20	33.00 ± 0.00	96.67 ± 2.89	6.97 ± 0.01
F21	<28.00	90.00 ± 5.00	7.00 ± 0.00

**Table 3:** Formulation composition of in situ gel loaded with ketoconazole NPs.

Formula-tion	Composition (% *w*/*v*)					
	Poloxamer 407	Hydroxypropyl Methylcellulose (HPMC)	Sodium Carboxymethyl Cellulose (SCMC)	Sodium Alginate	Ketoconazole NPs	Purified Water q.s to
F1	18.00				1.00	100.00
F2	17.00				1.00	100.00
F3	16.00				1.00	100.00
F4	18.00	0.50			1.00	100.00
F5	18.00	1.00			1.00	100.00
F6	18.00	2.00			1.00	100.00
F7	17.00	0.50			1.00	100.00
F8	17.00	1.00			1.00	100.00
F9	17.00	2.00			1.00	100.00
F10	18.00		0.05		1.00	100.00
F11	18.00		0.10		1.00	100.00
F12	18.00		0.2		1.00	100.00
F13	17.00		0.05		1.00	100.00
F14	17.00		0.10		1.00	100.00
F15	17.00		0.2		1.00	100.00
F16	18.00			0.50	1.00	100.00
F17	18.00			1.00	1.00	100.00
F18	18.00			2.00	1.00	100.00
F19	17.00			0.50	1.00	100.00
F20	17.00			1.00	1.00	100.00
F21	17.00			2.00	1.00	100.00

## Data Availability

Data can be made available on request to corresponding author.
